# Grey Wolf Optimizer with Behavior Considerations and Dimensional Learning in Three-Dimensional Tooth Model Reconstruction

**DOI:** 10.3390/bioengineering11030254

**Published:** 2024-03-05

**Authors:** Ritipong Wongkhuenkaew, Sansanee Auephanwiriyakul, Marasri Chaiworawitkul, Nipon Theera-Umpon, Uklid Yeesarapat

**Affiliations:** 1 Department of Computer Engineering, Faculty of Engineering, Biomedical Engineering Institute, Biomedical Engineering and Innovation Research Center, Chiang Mai University, Chiang Mai 50200, Thailand; ritipong.w@cmu.ac.th; 2 Department of Computer Engineering, Faculty of Engineering, Excellence Center in Infrastructure Technology and Transportation Engineering, Biomedical Engineering Institute, Biomedical Engineering and Innovation Research Center, Chiang Mai University, Chiang Mai 50200, Thailand; 3 Orthodontics and Pediatric Dentistry Department, Faculty of Dentistry, Chiang Mai University, Chiang Mai 50200, Thailand; marasri.chai@cmu.ac.th; 4 Department of Electrical Engineering, Faculty of Engineering, Biomedical Engineering Institute, Biomedical Engineering and Innovation Research Center, Chiang Mai University, Chiang Mai 50200, Thailand; nipon.t@cmu.ac.th; 5 Department of Computer Engineering, Faculty of Engineering, Chiang Mai University, Empress Dental Care Clinic, Chiang Mai 50200, Thailand; uklid_y@cmu.ac.th

**Keywords:** grey wolf optimizer (GWO), oral healthcare, iterative closest point (ICP), 3D image registration, hierarchical registration, 3D tooth model reconstruction

## Abstract

Three-dimensional registration with the affine transform is one of the most important steps in 3D reconstruction. In this paper, the modified grey wolf optimizer with behavior considerations and dimensional learning (BCDL-GWO) algorithm as a registration method is introduced. To refine the 3D registration result, we incorporate the iterative closet point (ICP). The BCDL-GWO with ICP method is implemented on the scanned commercial orthodontic tooth and regular tooth models. Since this is a registration from multi-views of optical images, the hierarchical structure is implemented. According to the results for both models, the proposed algorithm produces high-quality 3D visualization images with the smallest mean squared error of about 7.2186 and 7.3999 μm^2^, respectively. Our results are compared with the statistical randomization-based particle swarm optimization (SR-PSO). The results show that the BCDL-GWO with ICP is better than those from the SR-PSO. However, the computational complexities of both methods are similar.

## 1. Introduction

Three-dimensional reconstruction from multi-view images has been used in many applications, including in orthodontics. It is also used in diagnostic and treatment planning processes in adults and children with dental caries [1,2], especially dental caries that are a cause of chronic diseases in children [3]. With recent technology, e.g., laser or CT, there are several 3D reconstructions from multimodal images research works [4,5,6,7,8,9] from those technologies. However, in rural areas, there is limited access to these sophisticated devices and also insufficient oral healthcare [10]. In Thailand, the Dental Innovation Foundation, under royal patronage, has provided dental care access in rural communities for a long time. Due to limited access to sophisticated devices in those communities, taking multi-view teeth images inside children’s mouths is a very difficult task. Hence, multi-view teeth optical images are collected to be utilized in the 3D reconstruction system. In 3D reconstruction from multi-view images, there are several processes, including image registration, which is the transforming process of different sets of data into one coordinate system. There are existing works on 2D medical image registration [11,12,13,14,15,16,17,18,19,20,21,22,23,24,25,26]. Since point cloud coordinates are used in our 3D teeth reconstruction, the 3D registration is more proper. In the literature, there are several works on 3D registration [27,28,29,30,31] utilizing several features in the registration process, including point cloud coordinates representing the 3D shapes of objects [32,33,34,35,36,37]. These mentioned works used a variation swarm optimization (PSO) in the location matching between the source and target images. In the hope of improving the registration accuracy, there was study conducted on using the grey wolf optimizer (GWO) instead of PSO in 3D registration [38]. However, only rotation and/or translation were used in those matching locations. Hence, in our previous work [39], the statistical randomization-based particle swarm optimization (SR-PSO) algorithm with the iterative closet point (ICP) method was used to find the optimal affine transform (translation, scaling, rotation, and shearing (shortened from a shearing mapping that displaces each point in a fixed direction by an amount proportional to its signed distance from a given line parallel to that direction)) between teeth optical images.

In particular, a system with the 3D registration using a modified grey wolf optimizer that can reconstruct a 3D image from teeth optical images is developed. However, due to a research ethical approval requirement, we will not use real images taken from children. Hence, we postulate scanned images from two commercial tooth models and then create point cloud images [39]. To avoid premature convergence and to balance between exploration and exploitation, we modify the grey wolf optimization algorithm [40] with behavior considerations and dimensional learning strategies [41,42,43,44], called BCDL-GWO, to find the suitable affine transform between the source and target images. This can also enhance global and local searching and improve an ability to escape from a local optima. It has been shown in [41,42,43,44] that the BCDL-GWO is a good optimization tool when used to find suitable parameters in several applications, including engineering design problems (pressure vessel design, tension/compression spring design, and welded beam design problems), biomedical real-life problems (breast cancer and heart disease detection), and 14 real-world problems from the 2011 IEEE Congress on Evolutionary Computation. Furthermore, to refine the resulting registration, the iterative closet point (ICP) method [45,46] is used because of its ability to refine registered results [11,34,39]. In the final step, we reconstruct the 3D tooth models.

## 2. Registration Method

In this section, we will briefly review the 3D registration method used in this paper. The registration between two point cloud images (target (**P** = [***p****_i_*]*_M_*_× 4_, *M* is the number of target point cloud points) and source (**Q** = [***q****_j_*]*_N_*_× 4_, *N* is the number of source point cloud points) point cloud images) can be found by the following transformation:

(1) $$H^{*}=\arg\min f(H(Q),P)$$

where **H** is the geometry transform estimated by finding the nearest neighbor between a set of point pairs (***p****_j_* and ***q****_j_*) [46,47], and *f*(·) is an objective function (minimum distance error between two corresponding points). Hence, the mean squared error (MSE) can be used as *f*(·) to find a suitable H* as

(2) $$H^{*}=\arg\min_{H}\frac{1}{N}{\sum_{j=1}^{N}\left(q_{j}\cdot H^{T}-p_{j}\right)}^{2}$$

(3) $$p_{j}=\underset{p_{i}\in P}{\arg\min}\left\|q_{j}.H^{T}-p_{i}\right\|.$$

In this case, there are 15 unknown parameters, i.e., 3, 3, 3, and 6 parameters for scaling (**S**), translation (**T**), rotation (**R**), and shearing (**SH**), respectively [48]. To give a simpler equation, let *cox* = cos(*ϕ_x_*), *coy* = cos(*ϕ_y_*), *coz* = cos(*ϕ_z_*), *six* = sin(*ϕ_x_*), *siy* = sin(*ϕ_y_*), and *siz*=sin(*ϕ_z_*); then, the 3D transformation matrix **H** is computed as

**H** = **T** × **S** × **R** × **SH**, (4)

(5) $$H=\left[\begin{array}{cccc} 1 & 0 & 0 & t_{x}\\ 0 & 1 & 0 & t_{y}\\ 0 & 0 & 1 & t_{z}\\ 0 & 0 & 0 & 1 \end{array}\right]\left[\begin{array}{cccc} s_{x} & 0 & 0 & 0\\ 0 & s_{y} & 0 & 0\\ 0 & 0 & s_{z} & 0\\ 0 & 0 & 0 & 1 \end{array}\right]\left[\begin{array}{cccc} coy\times coz & -coy\times siz & siy & 0\\ six\times siy\times coz+cox\times siz & -six\times siy\times siz+cox\times coz & -six\times coy & 0\\ -cox\times siy\times coz+six\times siz & cox\times siy\times siz+six\times coz & cox\times coy & 0\\ 0 & 0 & 0 & 1 \end{array}\right]\left[\begin{array}{cccc} 1 & sh_{1} & sh_{2} & 0\\ sh_{3} & 1 & sh_{4} & 0\\ sh_{5} & sh_{6} & 1 & 0\\ 0 & 0 & 0 & 1 \end{array}\right]$$

Hence,

(6) $$H=\left[\begin{array}{cccc} a & d & g & t_{x}\\ b & e & i & t_{y}\\ c & f & j & t_{z}\\ 0 & 0 & 0 & 1 \end{array}\right],$$

where

(7) $$\begin{array}{l} a=s_{x}(coy\times coz)+sh_{3}s_{x}(-coy\times siz)+sh_{5}s_{x}(siy)\\ b=s_{y}(six\times siy\times coz+cox\times siz)+sh_{3}s_{y}(-six\times siy\times siz+cox\times coz)+sh_{5}s_{y}(-six\times coy)\\ c=s_{z}(-cox\times siy\times coz+six\times siz)+sh_{3}s_{z}(cox\times siy\times siz+six\times coz)+sh_{5}s_{z}(cox\times coy)\\ d=sh_{1}s_{x}(coy\times coz)+s_{x}(-coy\times siz)+sh_{6}s_{x}(siy)\\ e=sh_{1}s_{y}(six\times siy\times coz+cox\times siz)+s_{y}(-six\times siy\times siz+cox\times coz)+sh_{6}s_{y}(-six\times coy)\\ f=sh_{1}s_{z}(-cox\times siy\times coz+six\times siz)+s_{z}(cox\times siy\times siz+six\times coz)+sh_{6}s_{z}(cox\times coy)\\ g=sh_{2}s_{x}(coy\times coz)+sh_{4}s_{x}(-coy\times siz)+s_{x}(siy)\\ i=sh_{2}s_{y}(six\times siy\times coz+cox\times siz)+sh_{4}s_{y}(-six\times siy\times siz+cox\times coz)+s_{y}(-six\times coy)\\ j=sh_{2}s_{z}(-cox\times siy\times coz+six\times siz)+sh_{4}s_{z}(cox\times siy\times siz+six\times coz)+s_{z}(cox\times coy) \end{array}$$

It is worthwhile noting that *a* through *j* are non-rigid transformations resulting from the combination of scaling, shearing, and rotation properties.

To find the optimal H, the proposed grey wolf optimization algorithm with behavior considerations and dimensional learning strategies (BCDL-GWO) algorithm described in the following section is utilized. Table 1 shows the defined search space with 15-dimensional individuals in the swarm.

### 2.1. Overview of Grey Wolf Optimizer Algorithm (GWO)

The gray wolf optimizer (GWO) [40] algorithm is divided into five mathematical models, i.e., (1) social hierarchy, (2) encircling prey, (3) hunting prey, (4) attacking prey (exploitation), and (5) seeking prey (exploration). The wolves are first generated as a set of candidate solutions (search agents) by randomization. At each generation, the wolves, called omega (*ω*), are guided by their three leaders, named alpha (*α*), beta (*β*), and delta (*δ*), to find more favorable regions in search spaces while searching or hunting for prey. Let $X=\left\{x_{l}\left|l=1\ldots K\right.\right\};x_{l}=\left\{x_{lj}\left|j=1\ldots d\right.\right\}$ be a set of *K* search agents (individuals) with *d*-dimensional feature space. The encircling behavior of the *l*th grey wolf (**x***_l_*) around the *p*th prey (**x***_p_*) in the *j*th dimension at iteration (*t*) is

(8) $$D_{p}^{t}=\left|C_{p}^{t}\cdot x_{p}^{t}-x_{l}^{t}\right|$$

(9) $$x_{l}^{t}=x_{p}^{t}-A_{p}^{t}\cdot D_{p}^{t}$$

where $D_{p}^{t}$ is the distance between **x***_l_* and **x***_p_* at iteration *t*. The $A_{p}^{t}$ and $C_{p}^{t}$ are defined as

(10) $$A_{p}^{t}=2\times a\cdot r_{1}-a$$

(11) $$C_{p}^{t}=2\times r_{2}$$

where the components of **a** decrease linearly from 2 to 0 over the course of iterations. **r**_1_ and **r**_2_ are random vectors in [0, 1]. Therefore, each element in $A_{p}^{t}$ will be a random value in [−*a*, *a*], whereas that in $C_{p}^{t}$ will be a random value in [0, 2]. The position update equation of each individual will follow the 3 leaders, i.e., *α*, *β*, and *δ* represented by $x_{\alpha }^{t}$, $x_{\beta }^{t}$, and $x_{\beta }^{t}$, respectively.

(12) $$D_{\alpha }^{t}=\left|C_{\alpha }^{t}\cdot x_{\alpha }^{t}-x_{l}^{t}\right|,\;D_{\beta }^{t}=\left|C_{\beta }^{t}\cdot x_{\beta }^{t}-x_{l}^{t}\right|,\;\mathrm{and}\;D_{\delta }^{t}=\left|C_{\delta }^{t}\cdot x_{\delta }^{t}-x_{l}^{t}\right|$$

(13) $$x_{1}^{t}=x_{\alpha }^{t}-A_{\alpha }^{t}\cdot D_{\alpha }^{t},\;x_{2}^{t}=x_{\beta }^{t}-A_{\beta }^{t}\cdot D_{\beta }^{t},\;\mathrm{and}\;x_{3}^{t}=x_{\delta }^{t}-A_{\delta }^{t}\cdot D_{\delta }^{t}$$

then

(14) $$x_{l}^{t+1}=\frac{x_{1}^{t}+x_{2}^{t}+x_{3}^{t}}{3}$$

### 2.2. The Modified GWO Algorithm with Behavior Considerations and Dimensional Learning (BCDL-GWO)

The modified GWO with behavior considerations and dimensional learning is based on the idea of [41,42,43,44]. Firstly, we incorporate the Sine Cosine Algorithm (SCA) [41] in the alpha grey wolf to alleviate the unbalancing between exploration and exploitation and to help with the premature convergence by

(15) $$\begin{array}{l} D_{\alpha }^{t}=rand()\times \sin(rand())\times \left|C_{\alpha }^{t}\cdot x_{\alpha }^{t}-x_{l}^{t}\right|;\;if\;rand()<0.5\\ D_{\alpha }^{t}=rand()\times \cos(rand())\times \left|C_{\alpha }^{t}\cdot x_{\alpha }^{t}-x_{l}^{t}\right|;otherwise \end{array}$$

Each *j*th element of $C_{\alpha }^{t}$, $C_{\beta }^{t}$, and $C_{\delta }^{t}$ is modified following the method in [49] as

(16) $$\begin{array}{l} C_{\alpha ,j}^{t}=1+\left(2\times r_{3}-1\right)\times c^{2}\\ C_{\beta ,j}^{t}=1+\left(2\times r_{4}-1\right)\times c^{2}\\ C_{\delta ,j}^{t}=1+\left(2\times r_{5}-1\right)\times c^{2} \end{array}$$

where *r*_3_, *r*_4_, and *r*_5_ are the uniformly distributed random numbers in [0, 1]. ***c*** is decreasing linearly from 1 to 0 over the course of iterations as follows:

(17) $$c=c_{\max}-\left(c_{\max}-c_{\min}\right)\times \left(\frac{t-1}{T-1}\right);c_{\min}=0,c_{\max}=1$$

Therefore, each element in vector **C** is stochastically generated in [0, 2] in the first iteration and decreases to 1 at the final iteration. This process helps to provide a better exploration capability. The control vectors $A_{\alpha }^{t}$, $A_{\beta }^{t}$, and $A_{\delta }^{t}$ are calculated as in Equation (10) with *a* calculated by [50]

(18) $$a=2\times {\left(\frac{T-t}{T}\right)}^{\mu }$$

where *T* is the total number of iterations, and 0 < *μ* ≤ 2.

Our hypothesis is that the alpha grey wolf is the most important leader; hence, the updated position of each omega grey wolf is modified to [51]

(19) $$x_{l}^{t+1}=w_{1}^{t}x_{1}^{t}+w_{2}^{t}x_{2}^{t}+w_{3}^{t}x_{3}^{t};\;w_{1}^{t}+w_{2}^{t}+w_{3}^{t}=1$$

where

(20) $$\begin{array}{l} w_{1}^{t}=\cos\theta \\ w_{2}^{t}=0.5\sin\theta \cos\varphi \\ w_{3}^{t}=1-(w_{1}^{t}+w_{2}^{t})\\ \mathrm{with}\;\theta =\frac{2}{\pi }\cos^{-1}\left(\frac{1}{3}\right)\tan^{-1}(t),\varphi =0.5\tan^{-1}(t) \end{array}$$

From Equation (20), $w_{1}\geq w_{2}\geq w_{3}$. *w*_1_ is close to 1, and *w*_2_ and *w_3_* are close to 0 at the beginning. These values will finally be close to 1/3 in the last iteration.

Now, we are ready to incorporate real-life behavioral considerations into the algorithm [52] by discarding the wolves during the migration (prey searching) with low fitness values and allowing mating (crossover and mutation as in the genetic algorithm) to improve the pack’s diversity. However, in our GWO, the first half of the iteration is focused on the exploration behavior (when |*A*| > 1), whereas the remaining half is transformed into exploitation behavior (when |*A*| < 1). Hence, we applied the Lévy flights (LF) [42] and Random Opposition Learning (ROL) [43] to improve the pack’s diversity and to enhance the capability of the global and local search. The LF is also applied to each element *j* of the three leaders as

(21) $$\begin{array}{l} x_{1,j}^{levy}=\left(x_{\alpha ,j}^{t}-A_{\alpha ,j}^{t}\times D_{\alpha ,j}^{t}\right)+levy_{1,j}^{t}\\ x_{2,j}^{levy}=\left(x_{\beta ,j}^{t}-A_{\beta ,j}^{t}\times D_{\beta ,j}^{t}\right)+levy_{2,j}^{t}\\ x_{3,j}^{levy}=\left(x_{\delta ,j}^{t}-A_{\delta ,j}^{t}\times D_{\delta ,j}^{t}\right)+levy_{3,j}^{t} \end{array}$$

The LF is defined as

(22) $$levy_{i,j}^{t}=\eta_{j}\times \alpha_{j}⊕\frac{u}{{\left|v\right|}^{1/\beta }}\times \left(x_{i,j}^{t}-x_{\alpha ,j}^{t}\right)$$

where

(23) $$\begin{array}{l} \eta \sim N(0,1),u\sim N(0,\sigma_{u}^{2}),v\sim N(0,\sigma_{v}^{2})\\ \sigma_{u}={\left\{\frac{\Gamma (1+\beta )\sin(\pi \beta /2)}{\Gamma [(1+\beta )/2]\beta 2^{(\beta -1)/2}}\right\}}^{1/\beta },\sigma_{v}=1 \end{array}$$

and Γ is a standard grammar function. In the experiment, we set *β* to 1.5, and *α* decreases over time [53] as follows:

(24) $$\alpha_{j}=\frac{L/10}{\sqrt{t\times d}};\;L=\left(ub_{j}-lb_{j}\right)$$

with *lb_j_* and *ub_j_* are the lower and upper bound, respectively, of the search space in the *j*th dimension. To add in the ROL in the exploitation behavior [43], suppose ${\hat{x}}_{l}=\left\{{\hat{x}}_{l,j}\left|j=1\ldots d\right.\right\};{\hat{x}}_{l,j}\in \left[lb_{j},ub_{j}\right]$ be a *d*-dimensional vector with

(25) $${\hat{x}}_{l,j}=lb_{j}+ub_{j}-rand()\times x_{l,j}^{b}$$

where $x_{l}^{b}$ is the individual best [54] of the *l*th grey wolf. Now, we introduce a new candidate solution ($x_{l}^{BC}$) as

(26) $$x_{l,j}^{BC}=\left\{\begin{array}{l} \begin{array}{c} \left(w_{1}^{t}x_{1,j}^{t}+w_{2}^{t}x_{2,j}^{t}+w_{3}^{t}x_{3,j}^{t}\right)+levy_{j}^{t},\;if\left|A\right|>1\&rand()\leq 0.5\\ w_{1}^{t}x_{1,j}^{levy}+w_{2}^{t}x_{2,j}^{levy}+w_{3}^{t}x_{3,j}^{levy},\;\;\;if\left|A\right|>1\&rand()>0.5 \end{array}\\ {\hat{x}}_{l,j},\;\;\;\;\;\;\;\;\;\;\;if\left|A\right|\leq 1 \end{array}\right.$$

It is worth noting that the fitness function in the BCDL-GWO (*f*(·)) is the aforementioned MSE.

The new update position is

(27) $$x_{l}^{t+1}=\left\{\begin{array}{c} x_{l}^{BC},\;\;if\;f(x_{l}^{BC})<f(x_{l}^{t+1})\\ x_{l}^{t+1},\;\;\;\;\;otherwise \end{array}\right.$$

For the dimensional learning part [55], we suppose $x_{l}^{DL}=\left\{x_{l,j}^{DL}\left|j=1\ldots d\right.\right\}$. The distance between the current and the next position of $x_{l}^{t}$ is

(28) $$R_{l}^{t}=\left\|x_{l}^{t}-x_{l}^{t+1}\right\|$$

The neighborhood of $x_{l}^{t}$ is defined by

(29) $$N_{l}^{t}=\left\{x_{k}^{t}\left|D_{lk}^{t}\right.\leq R_{l}^{t}\right\}\;\mathrm{where}\;D_{lk}^{t}=\left\|x_{l}^{t}-x_{k}^{t}\right\|,x_{k}^{t}\in K$$

Hence, each *j*th element of $x_{l}^{DL}$ is calculated by

(30) $$x_{l,j}^{DL}=x_{l,j}^{t}+rand()\times \left(x_{n,j}^{t}-x_{r,j}^{t}\right)$$

where $x_{n}^{t}$ and $x_{r}^{t}$ are randomly selected from $N_{l}^{t}$ and search agents, respectively. Then, the updated position will be

(31) $$x_{l}^{t+1}=\left\{\begin{array}{l} x_{l}^{DL},\;\;if\;f(x_{l}^{DL})<f(x_{l}^{t+1})\\ x_{l}^{t+1}\;\;\;\mathrm{else} \end{array}\right.$$

Finally, the position of each grey wolf will only change if the next fitness value is better than the current one. Hence, the final update position equation will be

(32) $$x_{l}^{t+1}=\left\{\begin{array}{l} \;x_{l}^{t+1},\;\;\;\mathrm{if}\;f(x_{l}^{t+1})<f(x_{l}^{t})\\ x_{l}^{t},\;\;\;\;\mathrm{otherwise} \end{array}\right.$$

The BCDL-GWO algorithm is summarized as shown in Algorithm 1.
**Algorithm 1.** BCDL-GWO algorithm**Input:** *K* population size, maximum iterations *T*. **Output**: Optimal solution. **Initial:** *K* wolves, *t* = 0. **While** *t* ≤ *T* Evaluate fitness value $f(x_{l}^{t})$ for each wolf $x_{l}^{t}\;\forall l=1\;to\;K$. Find three best leaders, i.e., $x_{\alpha }^{t},x_{\beta }^{t},x_{\delta }^{t}$. Update individual best positions $x_{l}^{b}\;\forall l=1\;\mathrm{to}\;K$. Update $A_{p}^{t}$, and $C_{p}^{t}$ using Equations (10) and (11), respectively. **For** each wolf in GWO-SCA procedure   Update current position $x_{l}^{t+1}$ using Equation (19).   Evaluate fitness $f(x_{l}^{t+1})$. **End For** **For** each wolf in behavior considerations procedure   Generate new candidate solution $x_{l}^{BC}$ using Equation (26).   Evaluate fitness $f(x_{l}^{BC})$.   Update $x_{l}^{t+1}$ using Equation (27). **End For** **For** each wolf in dimensional learning procedure   Generate new candidate solution $x_{l}^{DL}$ using equations (30).   Evaluate fitness $f(x_{l}^{DL})$.   Update $x_{l}^{t+1}$ using Equation (31). **End For** Update $x_{l}^{t+1}$ using Equation (32). *t* = *t* + 1 **End While**

The BCDL-GWO optimal solution is selected from the global best in the last population. The iterative closest point algorithm (ICP) method, as in [45,55] with the Nelder–Mead simplex method [56], is utilized to fine-tune the registration results. Table 2 shows the parameters used in our experiment.

## 3. Experimental Results

**Figure 1:**
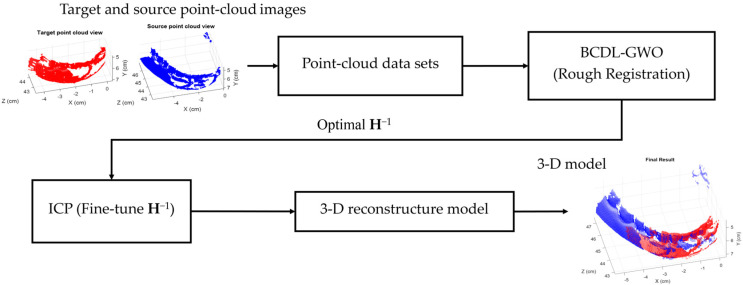
Three-dimensional reconstruction with BCDL-GWO.

The diagram of the 3D reconstruction with the optimal transformation matrix **H**^−1^ (transform from source point cloud to target point cloud) found by the BCDL-GWO is shown in Figure 1. To fine-tune the resultant **H**^−1^, the ICP method is used. Finally, the 3D tooth models are reconstructed based on the registered source and target point clouds.

**Figure 2:**
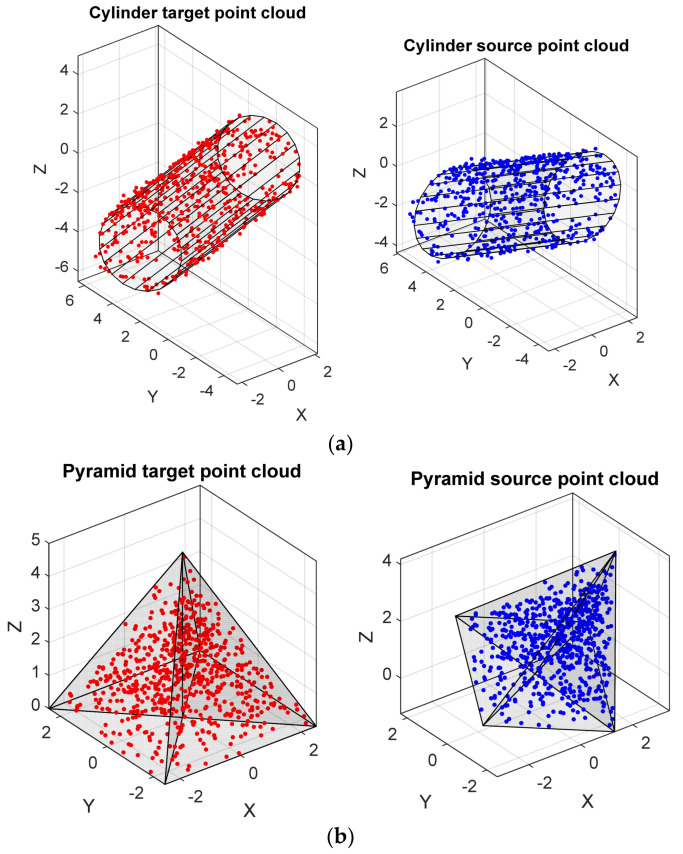
The original shape (target point cloud) and the 3D transformation (source point cloud) of (**a**) synthetic cylindrical and (**b**) synthetic pyramid shapes.

We first test our system on synthetic cylindrical and pyramid shapes as shown in Figure 2.

The transformation matrix used to generate from source to target point clouds for both shapes is

(33) $$H=\left[\begin{array}{cccc} 0.882050 & -0.285362 & -0.555884 & -0.061153\\ 0.225174 & 1.041540 & 0.181496 & 0.063487\\ 0.249299 & -0.413927 & 0.966936 & -0.163016\\ 0 & 0 & 0 & 1 \end{array}\right]$$

Hence, the **H**^−1^ that the BCDL-GWO needs to find is

(34) $$H^{-1}=\left[\begin{array}{rrrr} 0.901895 & 0.421703 & 0.439339 & 0.1\\ -0.143742 & 0.826257 & -0.237726 & -0.1\\ -0.294063 & 0.244980 & 0.819157 & 0.1\\ 0 & 0 & 0 & 1 \end{array}\right]$$

We compare the results from the BCDL-GWO with *μ* varied from 0.5 to 2.0 with a step size of 0.5 with those from our previous work (SR-PSO) [39]. Table 3 and Table 4 show the best registration MSEs results using the BCDL-GWO algorithm with and without refining the ICP method on the synthetic cylindrical and pyramid shapes, respectively. The best result on the synthetic cylindrical shape with MSE of 2.71 × 10^−27^ from BCDL-GWO is at *μ* = 0.5. Whereas that on the synthetic pyramid shape with MSE of 7.79 × 10^−20^ is at the same *μ*. The final best **H**^−1^ from both synthetic shapes is the same. Because the MSE is extremely small, both final best **H**^−1^ are the same as **H**^−1^ shown in Equation (34).

**Figure 3:**
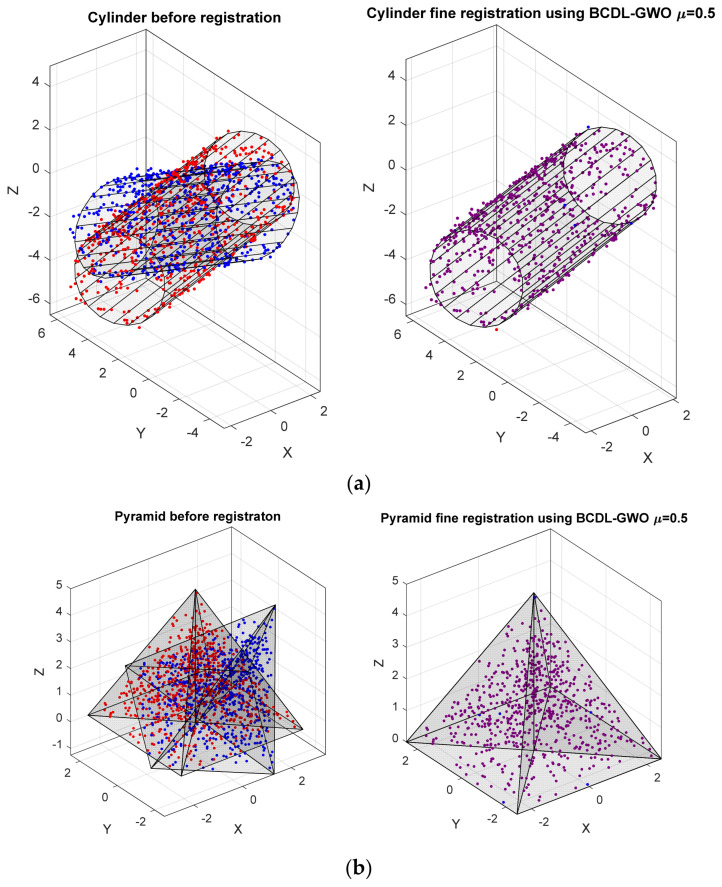
The final best registration using BCDL-GWO with *μ* = 0.5 of synthetic (**a**) cylindrical and (**b**) pyramid images.

The resulting registration of two cylindrical images and two pyramid images are shown in Figure 3a,b, respectively. It can be said that the best result from the BCDL-GWO is comparable with the best one from SR-PSO (*α* = 1.5). And when we look at the results from the BCDL-GWO with the other *μ*, those are better than that from the SR-PSO with the other *α*. To confirm this result, we also report the average ± standard deviation from several experiments of this algorithm on the same data set shown in Table 5 and Table 6 for the synthetic cylindrical and pyramid shapes, respectively.

We also compare the results from several experiments of both synthetic data sets shown in Table 7 and Table 8 with those achieved by the butterfly optimization algorithm (BOA) [57], Harris hawks optimization (HHO) [58], slime mold algorithm (SMA) [59], and whale optimization algorithm (WOA) [60], whereas each method has the best parameter setting. It can be seen that the result from BCDL-GWO without ICP is better than that from all compared methods with ICP. Hence, we can assume that our BCDL-GWO can escape local minima.

We also provide an indirect registration comparison of our BCDL-GWO with other methods without the utilization of swarm intelligence, i.e., Zhan et al. [33], Li et al. [61], and Du et al. [62], and as shown in Table 9. The results also confirm that our BCDL-GWO provides better results than its counterparts.

From the synthetic data set results, we are certain that the BCDL-GWO can be used in the tooth model 3D reconstruction. The regular tooth model and orthodontic tooth model from [39] were used in the experiment. For each model, six consecutive point cloud coordinate (*x*, *y*, *z*) views with an interval of 30 degrees are used in the experiment. Table 10 shows the information on the tooth point cloud data.

**Figure 4:**
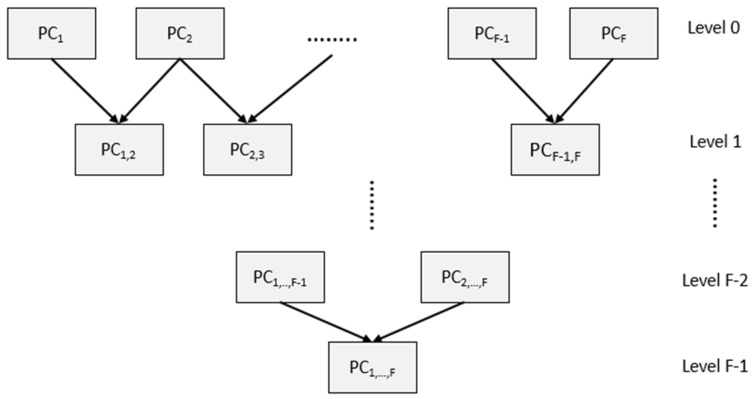
Hierarchical structure for multiple-views registration.

In this experiment, the size of the original image in each view was randomly sampled to 60% with the assumption that there was an overlap between each consecutive view. The voxel hull method [63,64,65] was used to select representative points inside the overlapping area. After that, the registration process with the parameter setting shown in Table 2 was implemented. Since there were six consecutive views, the hierarchical registration with *F* = 6 was used to increase the registration performance shown in Figure 4. The survival at each level was the best final registration result (BCDL-GWO algorithm with the ICP method), and that result proceeded to the next level of the hierarchical registration.

**Figure 5:**
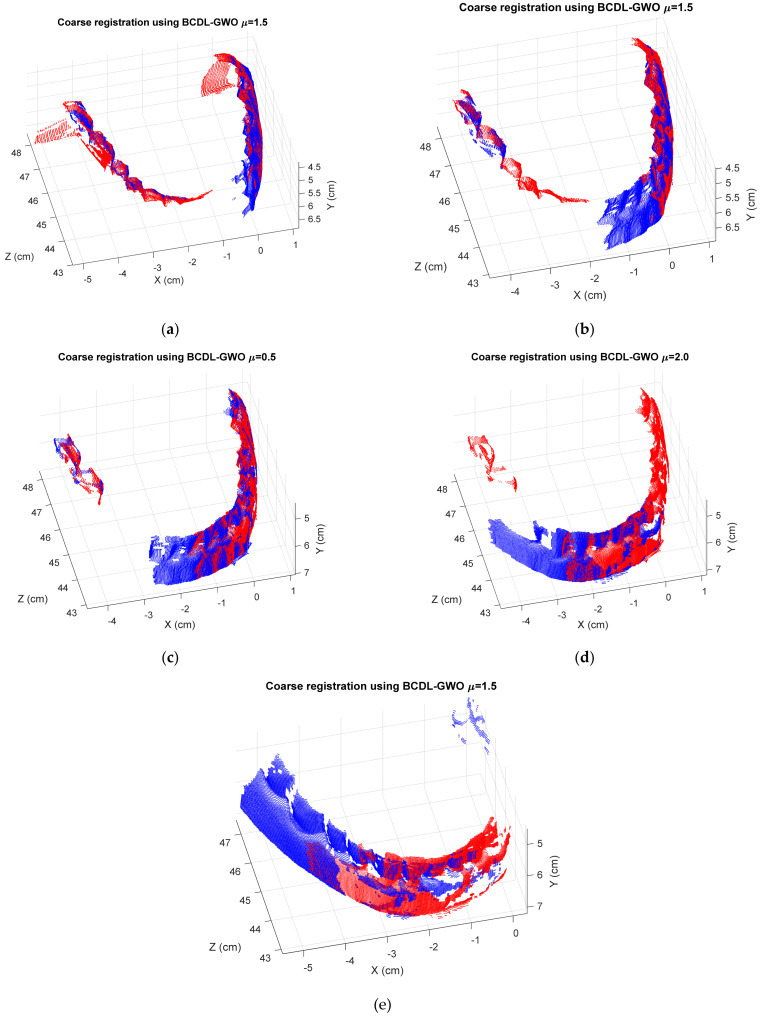
The best registration results of BCDL-GWO only for the following pairs: (**a**) 1 and 2; (**b**) 2 and 3; (**c**) 3 and 4; (**d**) 4 and 5; (**e**) 5 and 6.

**Figure 6:**
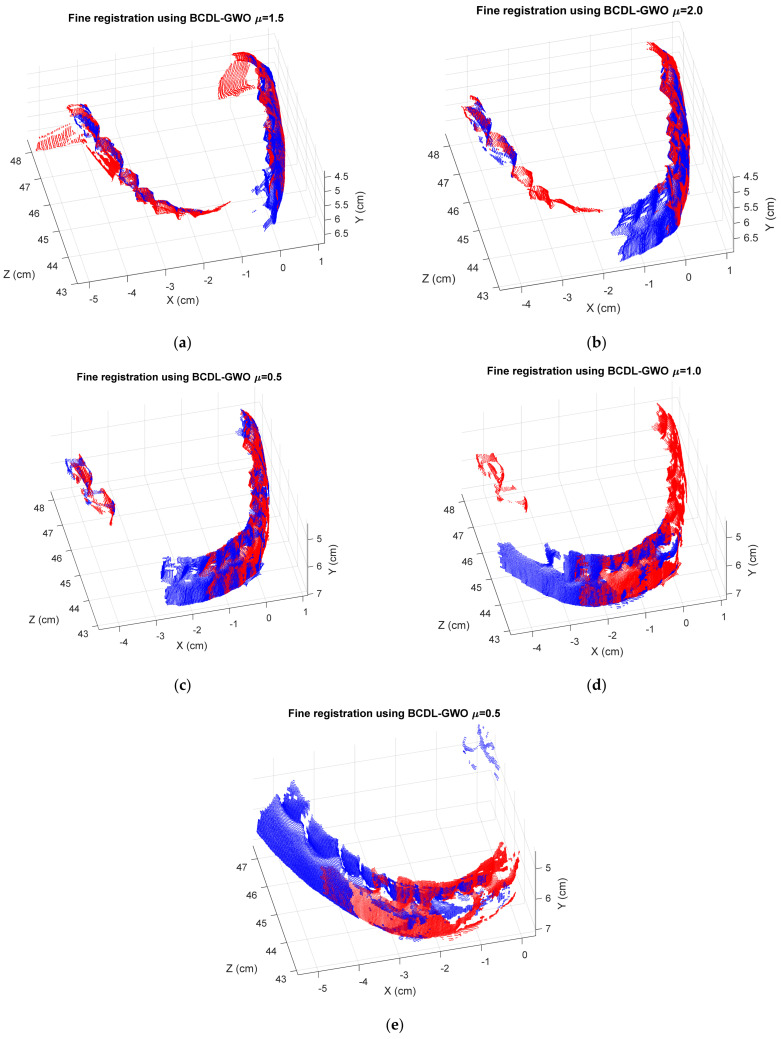
The best registration results of BCDL-GWO with ICP for the following pairs: (**a**) 1 and 2; (**b**) 2 and 3; (**c**) 3 and 4; (**d**) 4 and 5; (**e**) 5 and 6.

Table 11 shows the registration MSE results from the BCDL-GWO without the ICP of the regular tooth model at hierarchical level 1, whereas those with ICP are shown in Table 12. Figure 5 and Figure 6 show the best registration of each consecutive pair without and with ICP, respectively.

We compare our results with those from SR-PSO [39] as well. The MSEs of the regular tooth model at hierarchical level 1 are shown in Table 13. From the results, we can see that both methods provide comparable results. However, when we look at the MSEs of the final 3D reconstruction of the regular tooth model from six consecutive views shown in Table 14, we can see that the results from the best BCDL-GWO with ICP (7.2186 μm^2^ when *μ* = 0.5) are better than SR-PSO with ICP (7.3666 μm^2^).

**Figure 7:**
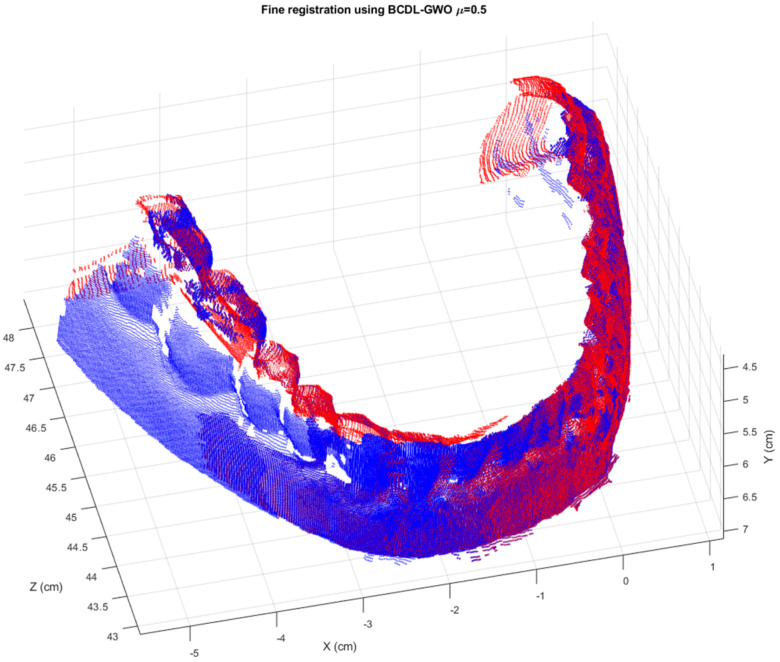
The final registration result of six consecutive views of the regular tooth model from the BCDL-GWO with the ICP.

The final registration of the regular tooth model is shown in Figure 7. We can see that the reconstruction result provides a good visualization.

**Figure 8:**
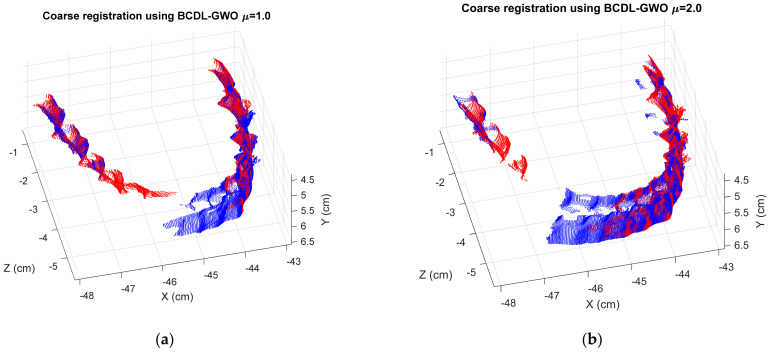

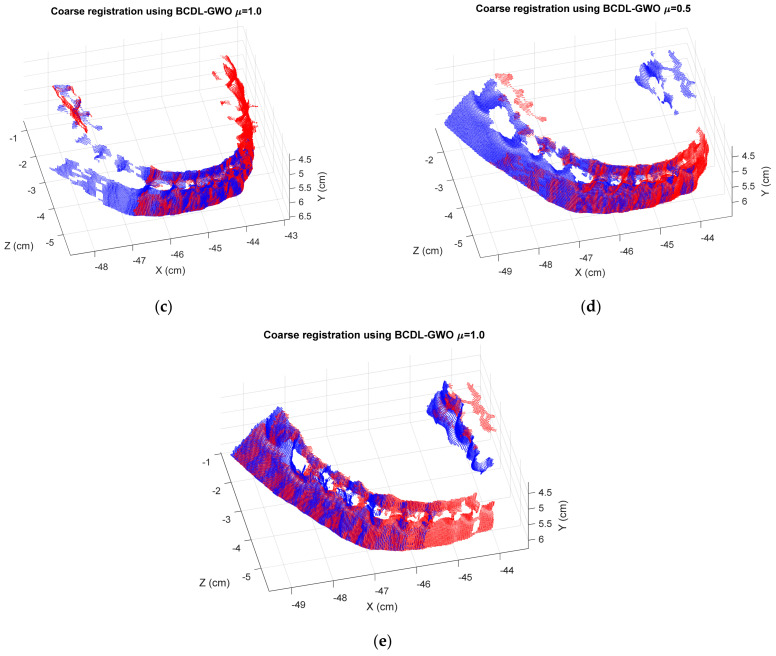
The best registration results of BCDL-GWO for the following pairs: (**a**) 1 and 2; (**b**) 2 and 3; (**c**) 3 and 4; (**d**) 4 and 5; (**e**) 5 and 6.

**Figure 9:**
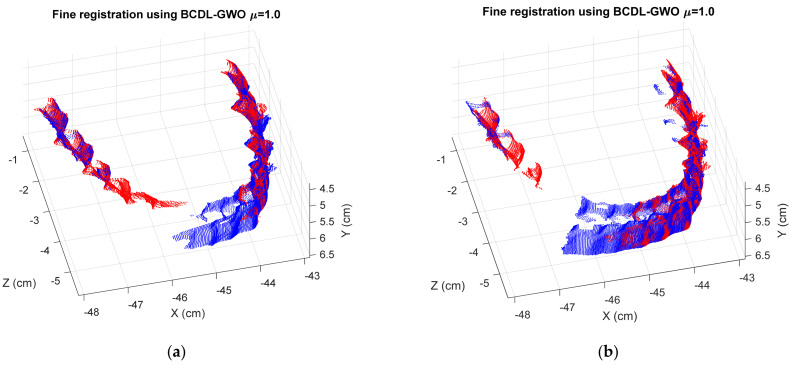

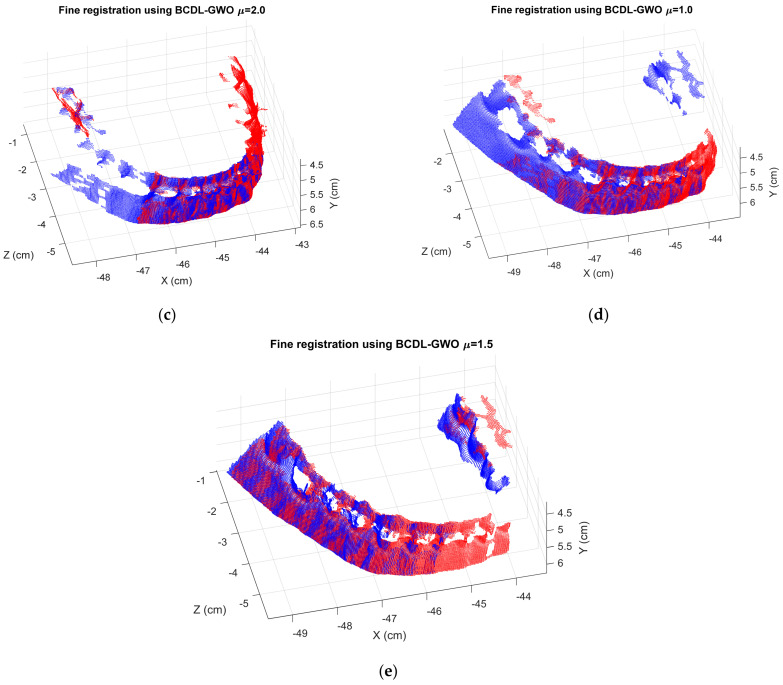
The best registration results of BCDL-GWO with ICP for the following pairs: (**a**) 1 and 2; (**b**) 2 and 3; (**c**) 3 and 4; (**d**) 4 and 5; (**e**) 5 and 6.

Finally, we implement the BCDL-GWO on the orthodontic tooth model to observe more experiments. The MSE registration results at hierarchical level 1 from the BCDL-GWO and BCDL-GWO with the ICP are shown in Table 15 and Table 16, respectively. Figure 8 and Figure 9 show the best registration results for each consecutive pair.

**Figure 10:**
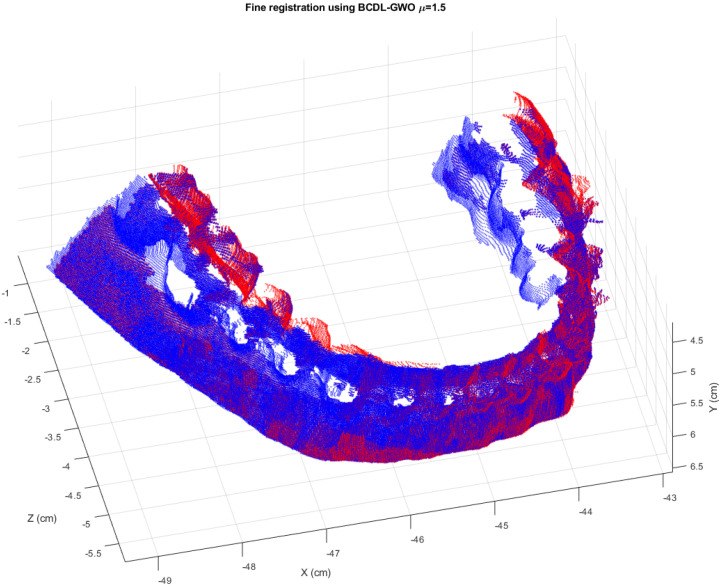
The final registration result of six consecutive views of the orthodontic tooth model from the BCDL-GWO with the ICP.

Again, we compare our MSEs on the registration results with those from the SR-PSO, as shown in Table 17. We can see that our proposed algorithm without the ICP method provides better registration results than the SR-PSO without the ICP method, except for a pair of 2 vs. 3. However, for the fine registration, our BCDL-GWO with ICP is comparable with those from the SR-PSO with ICP. But when we look at the final registration 3D orthodontic reconstruction model, as shown in Table 18, the results from the BCDL-GWO with ICP are better than the best results from the SR-PSO with ICP (7.4130 μm^2^). While the best result from the BCDL-GWO with ICP at *μ* = 1.5 is 7.3999 μm^2^. The final 3D reconstruction of the orthodontic tooth model is shown in Figure 10. We can see that the reconstruction result can still provide a good visualization.

To confirm that our method is good enough, we indirectly compare our results with those existing methods in the literature on the dental 3D registration data sets. However, those methods were performed on different data sets. The comparison results are shown in Table 19. Again, the results from our BCDL-GWO are better.

One might wonder what the computational complexity of BCDL-GWO is compared with the SR-PSO, BOA, HHO, SMA, and WOA shown in Table 20. To compute the complexity of the BCDL-GWO, we start with the population initialization step. Since there are *K* grey wolves in the population and each grey wolf is represented by a *D*-dimensional vector, the computational complexity in this step is *O*(*K* × *D*). For the control parameter step, the first operation is the GWO-SCA, which needs *O*(*K* × *D*). The next operation in this step is Equation (15), which will need *O*(*D*). Then, both the position update and fitness evaluation will need *O*(*K* × *D*). Hence, in this step, it is *O*(*K* × *D*). However, in the fitness comparison step, it will need *O*(*K*). The next step is the behavior consideration procedure. In this step, the new candidate solution calculation from Equation (26) will need *O*(*K* × *D*). The fitness calculation in this step will need *O*(*K* × *D*). The update position using Equation (27) will be *O*(*K* × *D*). Hence, in this step, the complexity will be *O*(*K* × *D*). Finally, in the dimensional learning procedure step, the distance calculation will need *O*(*K* × *D*). Equation (29) used in the search agents will need *O*(*K* × *D*^2^). Again, the position update needs *O*(*K* × *D*). However, in this step, the total complexity is *O*(*K* × *D*^2^). Since there are *T* iterations, the total complexity of the BCDL-GWO will be *O*(*T* × *K* × *D*^2^). For other algorithms, the complexities are calculated similarly. Table 20 shows the Big *O* of each step in each algorithm. The complexities of all algorithms are very similar. Even though the BCDL-GWO has a slightly higher complexity than the others, the tradeoff with the performance of our BCDL-GWO is still good.

## 4. Conclusions

To help in dental diagnostic and treatment planning in rural areas with limited access to sophisticated devices, a 3D reconstruction from multi-view optical images is needed. To provide a good 3D reconstruction, a good 3D registration process is required. In this paper, we developed the grey wolf optimization algorithm with behavior considerations and dimensional learning strategies (BCDL-GWO) with iterative closet point (ICP) to find the optimal affine transform in the 3D registration process. We compare the results with those from the statistical randomization-based particle swarm optimization (SR-PSO). We found that the final best result of BCDL-GWO with the ICP yields a mean squared error (MSE) of 7.2186 μm^2^ for 3D reconstruction from six consecutive views of the regular tooth model, whereas that of SR-PSO with the ICP method is 7.3666 μm^2^. The MSE of the BCDL-GWO with the ICP method is 7.3999 μm^2^ for the orthodontic tooth model, while the SR-PSO with the ICP provides 7.4130 μm^2^. We can say that the 3D reconstruction of the regular and orthodontic tooth models from the BCDL-GWO with ICP is better than the SR-PSO with ICP.

We also estimate the computational complexity of both the BCDL-GWO and the SR-PSO. We could say that they are comparable. However, from the nature of the BCDL-GWO, we can also say that it can cope with a premature convergence, an unbalance between exploration and exploitation, and finally, it increases a pack’s diversity.

Currently, there is only one research work involving the 3D model to assess dental caries [70]. This shows that there is a need for a 3D model for dental caries assessment. Hence, in future work, we plan to implement our algorithm in order to simulate dental caries for tooth defections in real situations.

## Figures and Tables

**Table 1:** Parameters boundaries in optimization process.

Parameters	Lower Bound	Upper Bound
*t_x_*, *t_y_*, *t_z_*	−1.5 (cm)	1.5 (cm)
*ϕ_x_*, *ϕ_y_*, *ϕ_z_*	−45 (deg)	45 (deg)
*s_x_*, *s_y_*, *s_z_*	0.8 (20% downscaling)	1.2 (20% upscaling)
*sh*_1_, *sh*_2_, *sh*_3, _*sh*_4_, *sh*_5_, *sh*_6_	−0.5 (cm)	0.5 (cm)

**Table 2:** BCDL-GWO parameters configuration.

Parameters	Symbols	Values
Search agents (wolves)	*K*	100
Iterations	*T*	2000
Control parameter *a*	*a*_0_	2
Control parameter *c*	*c*_0_	2
Nonlinear exponent indices	*μ*	0.5, 1.0, 1.5, 2.0
Lévy distribution	*β*	1.5

**Table 3:** The mean squared error (MSE) in pixels^2^ of the registration on the synthetic cylindrical shape.

**Research work in** [39]	*α*			
	*α* = 0.5	*α* = 1.0	*α* = 1.5	*α* = 2.0
Without ICP	7.68 × 10^−2^	9.23 × 10^−2^	**3.22 × 10^−31^**	9.68 × 10^−2^
With ICP	7.68 × 10^−2^	9.23 × 10^−2^	**3.22 × 10^−31^**	9.68 × 10^−2^
**BCDL-GWO**	*μ*			
	0.5	1.0	1.5	2.0
Without ICP	1.90 × 10^−9^	5.37 × 10^−11^	**3.46 × 10^−13^**	3.23 × 10^−12^
With ICP	**2.71 × 10^−27^**	7.35 × 10^−22^	4.11 × 10^−26^	3.41 × 10^−21^

**Table 4:** The mean squared error (MSE) in pixels^2^ of the registration on the synthetic pyramid shape.

**SR-PSO** [39]	*α*			
	*α* = 0.5	*α* = 1.0	*α* = 1.5	*α* = 2.0
Without ICP	1.14 × 10^−3^	1.18 × 10^−4^	**5.71 × 10^−30^**	1.70 × 10^−4^
With ICP	1.51 × 10^−17^	9.66 × 10^−18^	**5.71 × 10^−30^**	6.97 × 10^−18^
**BCDL-GWO**	*μ*			
	0.5	1.0	1.5	2.0
Without ICP	9.37 × 10^−10^	3.40 × 10^−10^	**1.96 × 10^−13^**	1.95 × 10^−12^
With ICP	**7.79 × 10^−20^**	1.23 × 10^−18^	1.63 × 10^−17^	6.51 × 10^−18^

**Table 5:** The average registration mean squared error (MSE) ± standard deviation in pixels^2^ on the synthetic cylindrical shape.

**SR-PSO** [39]	*α*			
	*α* = 0.5	*α* = 1.0	*α* = 1.5	*α* = 2.0
Without ICP	5.38 × 10^−2 ^ ± 3.73 × 10^−2^	4.66 × 10^−2 ^ ± 4.05 × 10^−2^	**3.84 × 10^−2 ^** **± 4.06 × 10^−2^**	5.56 × 10^−2 ^ ± 3.90 × 10^−2^
With ICP	5.38 × 10^−2 ^ ± 3.73 × 10^−2^	4.66 × 10^−2 ^ ± 4.05 × 10^−2^	**3.84 × 10^−2 ^** **± 4.06 × 10^−2^**	5.56 × 10^−2 ^ ± 3.90 × 10^−2^
**BCDL-GWO**	*μ*			
	0.5	1.0	1.5	2.0
Without ICP	2.91 × 10^−9 ^ ± 3.73 × 10^−9^	1.14 × 10^−10 ^ ± 1.7 × 10^−10^	**1.07 × 10^−11 ^** **± 1.67 × 10^−11^**	1.19 × 10^−11 ^ ± 2.26 × 10^−11^
With ICP	1.68 × 10^−19 ^ ± 4.48 × 10^−19^	**4.26 × 10^−22 ^** **± 1.03 × 10^−21^**	2.64 × 10^−19 ^ ± 8.31 × 10^−19^	5.69 × 10^−20 ^ ± 1.17 × 10^−19^

**Table 6:** The average registration mean squared error (MSE) ± standard deviation in pixels^2^ on the synthetic pyramid shape.

**SR-PSO** [39]	*α*			
	*α* = 0.5	*α* = 1.0	*α* = 1.5	*α* = 2.0
Without ICP	1.65 × 10^−4 ^ ± 3.68 × 10^−4^	4.92 × 10^−5 ^ ± 1.08 × 10^−4^	**1.92 × 10^−6 ^** **± 6.08 × 10^−6^**	1.71 × 10^−5 ^ ± 5.38 × 10^−5^
With ICP	2.83 × 10^−18 ^ ± 5.91 × 10^−18^	**4.90 × 10^−19 ^** **± 1.55 × 10^−18^**	5.71 × 10^−18 ^ ± 1.81 × 10^−17^	9.92 × 10^−18 ^ ± 2.79 × 10^−17^
**BCDL-GWO**	*μ*			
	0.5	1.0	1.5	2.0
Without ICP	2.30 × 10^−9 ^ ± 4.76 × 10^−9^	6.29 × 10^−10 ^ ± 9.14 × 10^−10^	2.49 × 10^−10 ^ ± 7.04 × 10^−10^	**7.35 × 10^−12 ^** **± 1.76 × 10^−11^**
With ICP	2.15 × 10^−16 ^ ± 6.56 × 10^−16^	3.79 × 10^−18 ^ ± 4.78 × 10^−18^	5.79 × 10^−18 ^ ± 9.57 × 10^−18^	**6.93 × 10^−19 ^** **± 2.05 × 10^−18^**

**Table 7:** The best average registration mean squared error (MSE) ± standard deviation in pixels^2^ on the synthetic cylindrical shapes.

	BOA [57]	HNO [58]	SMA [59]	WOA [60]	SR-PSO [39]	BCDL-GWO
Without ICP	1.08 × 10^−1 ^ ± 3.83 × 10^−3^	9.63 × 10^−2 ^ ± 7.49 × 10^−3^	4.78 × 10^−2 ^ ± 4.70 × 10^−2^	7.27 × 10^−2 ^ ± 1.99 × 10^−2^	3.84 × 10^−2 ^ ± 4.06 × 10^−2^	**1.07 × 10^−11 ^** **± 1.67 × 10^−11^**
With ICP	8.58 × 10^−2 ^ ± 1.35 × 10^−2^	8.84 × 10^−2 ^ ± 1.09 × 10^−2^	4.54 × 10^−2 ^ ± 4.79 × 10^−2^	6.16 × 10^−2 ^ ± 3.38 × 10^−2^	3.84 × 10^−2 ^ ± 4.06 × 10^−2^	**4.26 × 10^−22 ^** **± 1.03 × 10^−21^**

**Table 8:** The best average registration mean squared error (MSE) ± standard deviation in pixels^2^ on the synthetic pyramid shapes.

	BOA [57]	HNO [58]	SMA [59]	WOA [60]	SR-PSO [39]	BCDL-GWO
Without ICP	4.14 × 10^−2 ^ ± 2.07 × 10^−3^	4.05 × 10^−2 ^ ± 8.42 × 10^−3^	7.92 × 10^−3 ^ ± 6.26 × 10^−3^	3.77 × 10^−2 ^ ± 8.85 × 10^−3^	1.92 × 10^−6 ^ ± 6.08 × 10^−6^	**7.35 × 10^−12 ^** **± 1.76 × 10^−11^**
With ICP	3.26 × 10^−17 ^ ± 8.08 × 10^−17^	4.19 × 10^−17 ^ ± 1.21 × 10^−16^	5.39 × 10^−18 ^ ± 1.08 × 10^−17^	3.08 × 10^−16 ^ ± 8.65 × 10^−16^	**4.90 × 10^−19 ^** **± 1.55 × 10^−18^**	6.93 × 10^−19 ^ ± 2.05 × 10^−18^

**Table 9:** The indirect comparison of several registration data sets with other existing methods.

Data Sets	Objects	Root Mean Squared Error (RMSE)	
		Li et al. [61]	BCDL-GWO
SHOT	Super Mario	4.422 × 10^−1^	**4.06 × 10^−3^**
	Doll	4.9 × 10^−3^	**4.01 × 10^−3^**
	Duck	5.8 × 10^−3^	**5.23 × 10^−3^**
	Frog	4.1 × 10^−3^	**3.83 × 10^−3^**
	Peter Rabbit	**3.9 × 10^−3^**	4.05 × 10^−3^
	Squirrel	1.29 × 10^−2^	**3.17 × 10^−3^**
Stand ford		**Du et al. [62]**	**BCDL-GWO**
	Bunny	1.9935 × 10^−3^	**1.7912 × 10^−3^**
	Dragon	1.841 × 10^−3^	**1.7789 × 10^−3^**
	Happy Buddha	2.0950 × 10^−3^	**2.0279 × 10^−3^**
Cow and Feet		**Mean Squared Error (MSE)**	
		**Zhan et al. [33]**	**BCDL-GWO**
	Cow	1.43 × 10^−2^	**1.24 × 10^−22^**
	Feet of man	3.78 × 10^−16^	**2.13 × 10^−18^**

**Table 10:** Tooth data set information.

Model	Object View	Object Name	Number of Points
Regular tooth model	1	Img0	28,807
	2	Img1	28,970
	3	Img2	28,983
	4	Img3	25,809
	5	Img4	17,303
	6	Img5	21,739
	Total	Six views	151,592
Orthodontic tooth model	1	Img0	25,301
	2	Img1	25,772
	3	Img2	22,432
	4	Img3	17,167
	5	Img4	22,537
	6	Img5	24,148
	Total	Six views	137,357

**Table 11:** MSE of BCDL-GWO on the regular tooth model at hierarchical level 1.

View Pairs	MSE in Micrometer^2^			
	*μ* = 0.5	*μ* = 1.0	*μ* = 1.5	*μ* = 2.0
1 vs. 2	5.8775	6.7106	**5.8122**	5.8169
2 vs. 3	5.0568	4.9923	**4.9406**	4.9582
3 vs. 4	**5.3752**	5.4111	5.4080	5.3940
4 vs. 5	5.5786	5.5135	5.5601	**5.4953**
5 vs. 6	5.9304	5.7735	**5.7640**	5.7808

**Table 12:** MSE of the BCDL-GWO with ICP on the regular tooth model at hierarchical level 1.

View Pairs	MSE in Micrometer^2^			
	*μ* = 0.5	*μ* = 1.0	*μ* = 1.5	*μ* = 2.0
1 vs. 2	5.7533	5.7531	**5.7327**	5.7328
2 vs. 3	4.9278	4.9266	4.9291	**4.9265**
3 vs. 4	**5.3558**	5.3564	5.3854	5.3566
4 vs. 5	5.3860	**5.3289**	5.3320	5.3412
5 vs. 6	**5.7269**	5.7316	5.7331	5.7287

**Table 13:** MSE comparison on regular tooth model at level 1.

View Pairs	MSE in Micrometer^2^			
	Coarse Registration		Fine Registration	
	SR-PSO	BCDL-GWO	SR-PSO	BCDL-GWO
1 vs. 2	5.9300	**5.8122**	5.8628	**5.7327**
2 vs. 3	**4.8937**	4.9406	**4.8860**	4.9265
3 vs. 4	5.4310	**5.3752**	5.4017	**5.3558**
4 vs. 5	**5.2666**	5.4953	**5.1253**	5.3289
5 vs. 6	5.8166	**5.7640**	**5.6828**	5.7269

**Table 14:** MSE of the final registration of six consecutive views (μm^2^) for the regular tooth model (the best value is in bold).

SR-PSO with ICP	BCDL-GWO with ICP			
*α* = 1.5	*μ* = 0.5	*μ* = 1.0	*μ* = 1.5	*μ* = 2.0
**7.3666**	**7.2186**	7.2188	7.2209	7.2189

**Table 15:** MSE of BCDL-GWO on the orthodontic tooth model at hierarchical level 1.

View pairs	MSE in Micrometer^2^			
	*μ* = 0.5	*μ* = 1.0	*μ* = 1.5	*μ* = 2.0
1 vs. 2	5.5267	**5.5227**	5.6627	5.5413
2 vs. 3	6.2508	6.8335	6.2865	**6.1815**
3 vs. 4	5.5515	**5.3939**	5.6002	5.5869
4 vs. 5	**6.4284**	6.6458	6.4830	6.4737
5 vs. 6	6.1203	**5.3183**	5.4125	5.3231

**Table 16:** MSE of BCDL-GWO with ICP on the orthodontic tooth model at hierarchical level 1.

View Pairs	MSE in Micrometer^2^			
	*μ* = 0.5	*μ* = 1.0	*μ* = 1.5	*μ* = 2.0
1 vs. 2	5.5104	**5.5089**	5.5108	5.5147
2 vs. 3	6.1425	**6.1422**	6.1423	6.1422
3 vs. 4	5.2838	5.2842	5.2842	**5.2720**
4 vs. 5	6.3988	**6.3931**	6.4010	6.4010
5 vs. 6	5.2861	5.2863	**5.2804**	5.2820

**Table 17:** MSE comparison on orthodontic tooth model at hierarchical level 1.

View Pairs	MSE in Micrometer^2^			
	Coarse Registration		Fine Registration	
	SR-PSO	BCDL-GWO	SR-PSO	BCDL-GWO
1 vs. 2	5.5553	**5.5227**	5.5093	**5.5089**
2 vs. 3	**6.1613**	6.1815	6.1440	**6.1422**
3 vs. 4	5.4687	**5.3939**	**5.2706**	5.2720
4 vs. 5	6.5847	**6.4284**	6.3945	**6.3931**
5 vs. 6	5.3262	**5.3183**	**5.2801**	5.2804

**Table 18:** MSE of the final registration of six consecutive views (micrometer^2^) for the orthodontic-tooth model (the best value is in bold).

SR-PSO with ICP	BCDL-GWO with ICP			
*α* = 0.5	*μ* = 0.5	*μ* = 1.0	*μ* = 1.5	*μ* = 2.0
**7.4130**	7.4000	7.4008	**7.3999**	7.4001

**Table 19:** Indirect comparison results on dental 3D registration data sets.

Research Works	Methods	Objective Functions	Data Sets	Transformations	Registration Errors
Kalla et al. [66]	Downhill simplex method and deformation techniques	Matt’s Mutual Information (MMI)	CT images	Non-Rigid	**Pre-registered:** 0.546 ± 0.233 **Elastic-registered:** 0.666 ± 0.286
Kim et al. [67]	2D CNN and ICP	Curvature variance of neighbor (CVN)	CT images and 3D scanned models	Rigid	**Data set 1: ** 1.39 ± 2.67 mm **Data set 2: ** 2.37 ± 3.43 mm **Data set 3: ** 1.01 ± 2.10 mm
Kurniawan et al. [68]	ICP	Root Mean Squared Error (RMSE)	3D point clouds	Rigid	**Experiment 1:** 0.182 ± 0.032 mm **Experiment 2:** 0.187 ± 0.041 mm
Chung et al. [69]	CNN and Downhill simplex method	Clustered similarity	CT images and 3D scanned models	Rigid	**Surface-based error: ** 5.11 ± 2.54 mm **Landmark-based error: ** 1.80 ± 0.84 mm
Our proposed method	BCDL-GWO and ICP	Mean Squared Error (MSE)	3D point clouds	Non-Rigid	**Tooth model 1:** 7.22 × 10^−3^ mm **Tooth model 2:** 7.39 × 10^−3^ mm

**Table 20:** Computational time complexities of BCDL-GWO and SR-PSO.

Process	Time Complexities					
	BCDL-GWO	SR-PSO [39]	BOA [57]	HHO [58]	SMA [59]	WOA [60]
Initialization	*O*(*K* × *D*) *	*O*(*K* × *D*)	*O*(*K* × *D*)	*O*(*K* × *D*)	*O*(*K* × *D*)	*O*(*K* × *D*)
Control parameter calculations	*O*(*K* × *D*)	*O*(*K* × *D*)	*O*(*K*)	*O*(*K*)	*O*(*K* × *D*)	*O*(*K* × *D*)
Position update steps	*O*(*K* × *D*)	*O*(*K* × *D*)	*O*(*K* × *D*)	*O*(*K* × *D*)	*O*(*K* × *D*)	*O*(*K* × *D*)
New candidate generation steps	*O*(*K* × *D^2^*)	*O*(*K* × *D*)	*-*	*-*	*-*	*-*
Fitness evaluations	*O*(*K* × *D*)	*O*(*K* × *D*)	*O*(*K* × *D*)	*O*(*K* × *D*)	*O(K* × *D*)	*O(K* × *D*)
Fitness comparisons	*O*(*K*)	*O*(*K*)	*O(K)*	*O(K)*	*O(KlogK*)	*O(K)*

* *K* denotes population size, and *D* indicates the number of dimensions in search spaces.

## Data Availability

No new data were created or analyzed in this study. Data sharing is not applicable to this article.
